# Mechanism-Centric Approaches for Biomarker Detection and Precision Therapeutics in Cancer

**DOI:** 10.3389/fgene.2021.687813

**Published:** 2021-08-02

**Authors:** Christina Y. Yu, Antonina Mitrofanova

**Affiliations:** ^1^Department of Biomedical and Health Informatics, School of Health Professions, Rutgers, The State University of New Jersey, Newark, NJ, United States; ^2^Rutgers Cancer Institute of New Jersey, Rutgers, The State University of New Jersey, New Brunswick, NJ, United States

**Keywords:** biomarkers, treatment response, precision medicine, predictive models, mechanism-centric approaches

## Abstract

Biomarker discovery is at the heart of personalized treatment planning and cancer precision therapeutics, encompassing disease classification and prognosis, prediction of treatment response, and therapeutic targeting. However, many biomarkers represent passenger rather than driver alterations, limiting their utilization as functional units for therapeutic targeting. We suggest that identification of driver biomarkers through mechanism-centric approaches, which take into account upstream and downstream regulatory mechanisms, is fundamental to the discovery of functionally meaningful markers. Here, we examine computational approaches that identify mechanism-centric biomarkers elucidated from gene co-expression networks, regulatory networks (e.g., transcriptional regulation), protein–protein interaction (PPI) networks, and molecular pathways. We discuss their objectives, advantages over gene-centric approaches, and known limitations. Future directions highlight the importance of input and model interpretability, method and data integration, and the role of recently introduced technological advantages, such as single-cell sequencing, which are central for effective biomarker discovery and time-cautious precision therapeutics.

## Introduction

In the past two decades, the advancement of high-throughput technologies has led to the discovery of genomic, transcriptomic, and epigenomic modalities involved in cancer initiation, progression, and treatment response. Multiple groups have started to effectively utilize molecular data produced by high-throughput oncology experiments to identify biomarkers of progression and therapeutic response in cancer patients (Sorlie et al., 2001; Zhang et al., 2001; van’t Veer et al., 2002; Zhan et al., 2002, 2006; Sotiriou et al., 2003; Ayers et al., 2004; Allen et al., 2006; Jain et al., 2009; Lim et al., 2009; Petty et al., 2009; Zhao et al., 2009; Carro et al., 2010; Lefebvre et al., 2010; Shaughnessy et al., 2011; Bae et al., 2013; Aytes et al., 2014, 2018; Mitrofanova et al., 2015; Robinson et al., 2015; Wang et al., 2016; Giulietti et al., 2017; Heng et al., 2017; Hoadley et al., 2018; Abida et al., 2019; Epsi et al., 2019; Arriaga et al., 2020; Panja et al., 2020; Rahem et al., 2020). Yet, our understanding of the mechanisms involving these modalities, their upstream regulation, and effective therapeutic targeting remains incomplete.

A biomarker is an objective measure (e.g., classically a genomic/transcriptomic/epigenomic alteration, gene, protein, metabolite, or their groups), typically used to predict the incidence of disease, its progression, or treatment outcome (Strimbu and Tavel, 2010; McDermott et al., 2013). In the context of oncology, biomarkers are classically used for cancer risk assessment and screening, tumor staging, disease recurrence, selection of initial therapy, alternative therapy choices, and monitoring for therapeutic toxicities (Ludwig and Weinstein, 2005). While employed in clinical use, the existing biomarkers are still sparse and suffer from issues of reproducibility and heterogeneity, alongside a lack of understanding of their underlying regulatory mechanisms (Ludwig and Weinstein, 2005; Boutros, 2015).

One of the reasons for such a knowledge gap is the fact that the majority of biomarkers are identified from *gene-centric* approaches (we will refer to gene/protein/metabolite etc.,-centric approaches as gene-centric approaches for simplicity), where either a specific gene is investigated (based on previous biological assumptions) or a gene(s) is selected based on differential behavior without connection to the upstream and downstream molecular mechanisms. Gene-centric findings are often limited in mechanistic interpretability and connectivity to other molecular processes, positioning such biomarkers as passengers, rather than drivers, of the biological process and thus are often dataset specific (Michiels et al., 2005; Chng et al., 2016).

In classical gene-centric approaches, genes (without their connections to one another or underlying mechanisms) are utilized as inputs into white- and black-box statistical and machine learning models, which have been successfully applied to identify gene-centric markers in breast cancer (van’t Veer et al., 2002; Wang et al., 2005; Zhang et al., 2013), lung cancer (Beer et al., 2002), multiple myeloma (Shaughnessy et al., 2007; Kuiper et al., 2012), colon cancer (Zhang et al., 2001; Yan et al., 2012), and prostate cancer (Garzotto et al., 2005; Erho et al., 2013), among many others. It is important to note that in white-box models (e.g., linear regression and decision trees) the relationship between input variables (i.e., genes) and output variables (i.e., disease outcomes) is understandable/explainable as they often identify linear or monotonic relationships (Zhang et al., 2001; Garzotto et al., 2005; Rosenfeld et al., 2008; Huo et al., 2017; Panja et al., 2018). On the other hand, black-box models (e.g., neural networks, gradient boosting, or ensemble models such as random forest) are able to capture non-linear/non-monotonic relationships, yet often suffer from model interpretability and subsequent limited clinical adoption (Wang et al., 2009; Ayer et al., 2010; Zhang et al., 2013). Even though both white- and black-box learning are excellent tools for predictive modeling, they mostly capture associative relationships when applied as gene-centric approaches and often miss the complexity of mechanisms inherent in biological systems, especially in the context of cancer.

**FIGURE 1:**
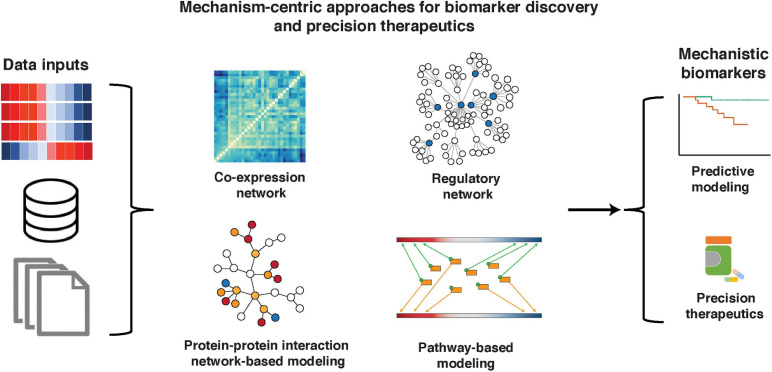
Mechanism-centric approaches in biomarker discovery and precision therapeutics. A variety of data, including single- and multi-omic sources, knowledge bases, and phenotype/clinical information, can be used as inputs to mechanism-centric approaches to identify functional biomarkers of disease and therapeutic response. We describe mechanism-centric methods that are based on co-expression networks, regulatory networks, PPI networks, and molecular pathways.

Several groups have addressed this problem by developing biomarker discovery methods based on *mechanism-centric* approaches, which are not focused on single genes and take into account complex mechanisms implicated in cancer initiation, progression, and treatment response. In this review, we will discuss the mechanism-centric approaches based on construction and mining of co-expression networks (Freeman, 1977; Zhang and Horvath, 2005; Zhang and Huang, 2014; Han et al., 2016), regulatory networks (Basso et al., 2005; Lefebvre et al., 2010; Alvarez et al., 2016; Dhingra et al., 2017), protein–protein interaction (PPI) networks (Chuang et al., 2007), and molecular pathways (Epsi et al., 2019; Rahem et al., 2020; Figure 1). Through an in-depth understanding of upstream and downstream molecular mechanisms, such techniques open a door for the discovery of functionally interpretable molecular drivers (rather than passengers) and potential targets for precision therapeutics.

## Mechanism-Centric Computational Approaches for Biomarker Discovery

### Gene Co-expression Network Analysis

Gene co-expression networks define groups of genes that show similar/related expression patterns across an entire dataset. Highly associated genes are clustered together into modules, with the underlying rationale that co-expressed genes are likely to be co-regulated. We depict two methods, weighted gene co-expression network analysis (WGCNA) (Langfelder and Horvath, 2008) and local maximal Quasi-Clique Merger (lmQCM) (Zhang and Huang, 2014), for network construction and module detection. Identified modules are defined as tightly connected groups of genes (potentially protein/gene complexes), which are then associated with clinical features to determine functionally relevant molecular structures. We also describe methods to mine such co-expression networks that include condition-specific network mining (Han et al., 2016), eigengene association (Alter et al., 2000; Zhang and Horvath, 2005), and network connectivity/hub analysis (Freeman, 1977).

#### Network Construction: WGCNA and lmQCM

**FIGURE 2:**
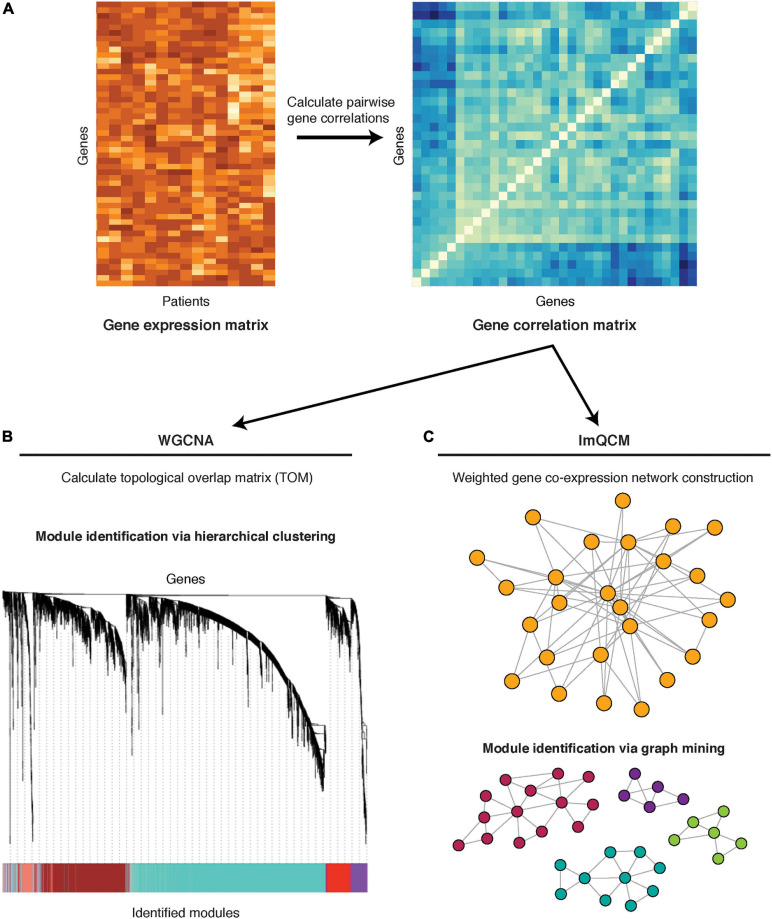
Co-expression network methods: WGNCA and lmQCM. **(A)** Pairwise gene correlations are calculated from gene expression (microarray or RNA-seq) data. **(B)** The co-expression matrix is transformed into a topological overlap matrix and subjected to hierarchical clustering for module identification. A cluster dendrogram is shown, with different gene modules identified by the color bar on the bottom. **(C)** The co-expression matrix is used to construct a network, with genes as nodes and the correlation co-efficient between any two genes as the edge weight. Module identification is achieved through a greedy search for highly correlated subnetworks.

In general, co-expression network construction is based on a similarity matrix that describes the measure of association between a gene to all other genes (the simplest of similarity measures being correlation) (Figure 2A). An undirected network is constructed from the similarity matrix and is comprised of nodes denoting genes and edges denoting the associations (e.g., correlation) between genes.

One of the most well-known methods for gene co-expression network reconstruction is WGCNA, which was one of the earliest methods that proposed using weighted networks (Figure 2B; Zhang and Horvath, 2005). The advantage of weighted, compared to unweighted, network construction is the ability to assign meaningful weights to relationships/edges, which eliminates a need for threshold assignment and prevents information loss. WGCNA calculates correlation between pairs of genes and transforms the correlation measure into a topological overlap measure in order to minimize effects of noise and spurious associations. The resulting matrix is subjected to hierarchical clustering to determine groups of co-expressed genes, also referred to as gene modules. An R package for WGCNA is freely available (Langfelder and Horvath, 2008).

Because WGCNA module identification is based on hierarchical clustering, genes cannot be assigned to multiple modules, exposing WGCNA’s limitation since many genes participate in multiple biological processes and often perform multiple functions. An alternative weighted co-expression method which allows genes to have multiple co-memberships in different modules is lmQCM (Figure 2C; Zhang and Huang, 2014). The lmQCM algorithm identifies densely connected subnetworks (i.e., quasi-cliques) using a greedy search algorithm which allows module overlaps (Ou and Zhang, 2007). In addition to allowing genes to be assigned to multiple modules, lmQCM can also identify smaller modules, which can highlight more specific and interpretable biological connections as compared to much larger modules of WGCNA that frequently contain over a thousand genes (Zhang and Huang, 2014; Yu et al., 2019). This algorithm is freely available as an R package^1^ and a web-tool (Huang et al., 2021).

#### Network Mining: Centered Concordance Index, Eigengenes, and Hubs

Co-expression networks can be mined to determine the functional significance of their modules or identify functionally relevant genes. Here, we discuss two techniques for module mining [Centered Concordance Index (CCI) (Han et al., 2016) and eigengenes (Alter et al., 2000; Horvath and Dong, 2008)] and two techniques to identify hub genes [intramodular connectivity (Zhang and Horvath, 2005) and betweenness centrality (Freeman, 1977)].

Centered Concordance Index has been developed to identify modules specific to each condition/phenotype. In particular, the CCI evaluates the concordance of gene expression profiles within a module based on singular value decomposition and is used to identify modules that are highly co-expressed in one condition over another (Han et al., 2016). Han et al. (2016) and Yu et al. (2019), respectively, identified several gene modules specific to lung adenocarcinoma and multiple myeloma precursors compared to non-cancer controls. The CCI is useful in identifying modules specific to phenotype conditions but has yet to be used to associate modules with continuous outcomes.

The eigengene approach transforms modules into weighted vectors, which mathematically correspond to their contribution to the first principal component in principal component analysis (Alter et al., 2000; Horvath and Dong, 2008). Eigengenes are then able to be associated with clinical features (including continuous outcomes) using correlation/association measures. For instance, Liu et al. (2015a) used the eigengene approach to identify two modules significantly associated with poor outcome in ER + breast cancer patients treated with tamoxifen. Liu et al. (2015b) and Zhang J. et al. (2020) associated module eigengenes derived from breast cancer patient data with clinical features such as survival status, tumor metastasis, and chemotherapy response. Han et al. (2019) identified module eigengenes strongly associated with patient survival in neuroblastoma.

The translational applicability of modules can be hampered by their relatively large size and might benefit from identification of hub genes within modules. Several measures have been developed to identify hubs, including intramodular connectivity and betweenness centrality. In particular, intramodular connectivity for gene *i* is defined as the sum of edge weights between gene *i* and the other genes in the module (Zhang and Horvath, 2005). Genes with the highest connectivity are considered hub genes and have been shown to play key roles in maintaining essential cellular functions (Jeong et al., 2001) and significantly associated with patient survival in breast cancer (Liu et al., 2015a; Tang et al., 2018; Jia et al., 2020; Tian et al., 2020; Zhang J. et al., 2020), glioblastoma (Horvath et al., 2006; Yang et al., 2018; Tang et al., 2019), hepatocellular carcinoma (Hu et al., 2020; Song et al., 2020), and pancreatic ductal adenocarcinoma (Giulietti et al., 2016), among others. Some of these findings have been experimentally validated, such as the ASPM hub gene in glioblastoma (Horvath et al., 2006) and FAM171A1, NDFIP1, SKP1, and REEP5 hub genes in breast cancer (Tian et al., 2020).

An alternative measure to identify hub genes is betweenness centrality, which is a network topology metric used to identify central nodes in a graph based on a shortest paths algorithm (Freeman, 1977). The betweenness centrality of gene *i* is a measure of the number of shortest paths connecting any two genes which pass through *i*. Genes with the highest betweenness scores are considered hubs and are believed to play an important role in information transfer within the network. For instance, Wang et al. analyzed modules with the betweenness centrality measure to identify eight hub genes that were significantly associated with overall survival in breast cancer patients (Wang C. C. N. et al., 2019).

### Regulatory Network Analysis

In recent years, molecular regulatory networks have received much attention from the scientific community due to their ability to capture complexity of molecular interactions present in cancer context-specific tissues (Butte and Kohane, 2000; Butte et al., 2000; Friedman et al., 2000; Basso et al., 2005; Margolin et al., 2006a,b; Werhli and Husmeier, 2007; Huynh-Thu et al., 2010; Lefebvre et al., 2010; Aytes et al., 2014). Regulatory networks define regulatory relationships between regulators (e.g., transcriptional regulators, splicing regulators, post-translational regulators, etc.), and their potential targets (e.g., genes, proteins, etc.). Such regulatory relationships provide key information about upstream and downstream regulations to infer cellular mechanisms for creating potential causal models of disease and outperform co-expression networks in their interpretability and functionally relevant determinants. Several methods have tackled reconstruction of regulatory networks using mutual information (Butte and Kohane, 2000; Basso et al., 2005; Margolin et al., 2006a), Bayesian networks (Friedman et al., 2000; Werhli and Husmeier, 2007), and regression trees (Huynh-Thu et al., 2010), to name a few. Readers are encouraged to consult the following reviews for a comprehensive overview of the different computational underpinnings employed in regulatory network analysis (Markowetz and Spang, 2007; Karlebach and Shamir, 2008; Hecker et al., 2009; Lee and Tzou, 2009; Emmert-Streib et al., 2014). Here, we focus on transcriptional [Algorithm for the Reconstruction of Gene Regulatory Networks (ARACNe) (Margolin et al., 2006a)] and multi-omic [RegNetDriver (Dhingra et al., 2017)] regulatory networks and their mining [i.e., Master Regulator Inference Algorithm (MARINa) (Lefebvre et al., 2010), Virtual Inference of Protein-activity by Enriched Regulon analysis (VIPER) (Alvarez et al., 2016), etc.] in the context of cancer biomarker studies.

#### Transcriptional Regulatory Networks

The role of transcriptional regulation has been widely studied in cancer, including discovery of MYC (Gabay et al., 2014), Sox2 (Boumahdi et al., 2014), and the FOXO family (Jiramongkol and Lam, 2020) as important players in cancer initiation and progression. Transcriptional regulatory networks depict interactions between transcription factors (TFs)/co-factors (co-TFs) and their transcriptional targets, allowing the study of differential behavior in transcriptional machinery that govern oncogenic process.

##### Network construction: ARACNe

One of the most known and widely experimentally validated methods for transcriptional network reconstruction is ARACNe (Margolin et al., 2006a,b). This information-theoretic algorithm utilizes tissue-specific gene expression profiles to estimate pairwise mutual information between expression levels of TFs/co-TFs and expression levels of their potential (activated or repressed) targets. The advantage of using mutual information to measure such relationships lies in its ability to measure not only linear (which would be captured for example by the Pearson correlation) or monotonic (which would be captured for example by Spearman correlation) relationships, but also non-linear associations. Another novelty in transcriptional network reconstruction is introduced by the data processing inequality, which eliminates any “indirect” regulatory relationship through the principle that mutual information on the indirect path cannot exceed mutual information on any part of the direct path. Data processing inequality results in a regulatory network that includes primarily direct TF/co-TF-target interactions. ARACNe has been widely applied to several normal physiological and pathological conditions, including B-cell interactome (Basso et al., 2005), breast cancer (Lim et al., 2009; Remo et al., 2015; Walsh et al., 2017), prostate cancer (Aytes et al., 2014), colorectal cancer (Bae et al., 2013; Cordero et al., 2014; Sanz-Pamplona et al., 2014; Eskandari et al., 2018), glioma (Carro et al., 2010), T-cell acute lymphoblastic leukemia (Palomero et al., 2006), and multiple myeloma (Agnelli et al., 2011), among others. Software for ARACNe is freely available for download.^2^

##### Network mining: MARINa and VIPER

**FIGURE 3:**
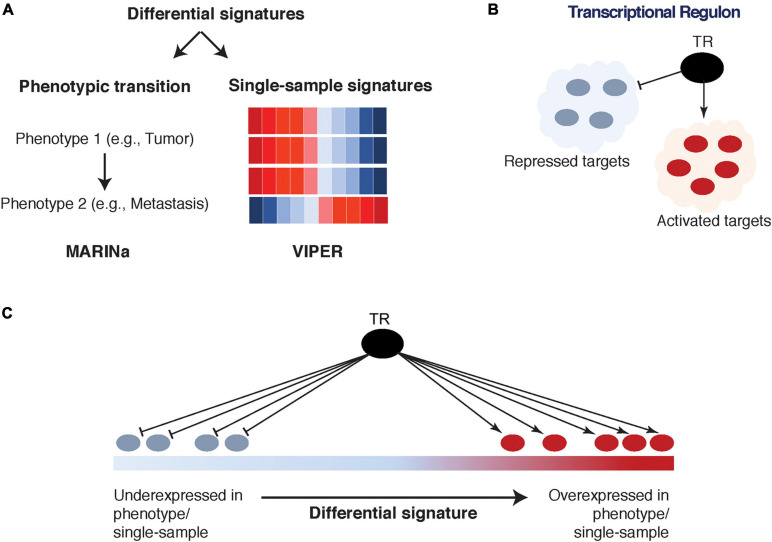
Interrogation of transcriptional regulatory networks: Master Regulator Inference Algorithm (MARINa) and Virtual Inference of Protein-activity by Enriched Regulon analysis (VIPER). **(A)** A differential signature is defined between two phenotypes of interest (left) as input to MARINa; or on a single-sample level (right) as input to VIPER. **(B)** The transcriptional regulon is identified from Algorithm for the Reconstruction of Gene Regulatory Networks (ARACNe) tissue-specific transcriptional regulatory network, which includes a transcriptional regulator (TR) and its activated and repressed targets. **(C)** The activated and repressed targets of the regulon are mapped onto the corresponding signature and used to determine the TR’s transcriptional activity.

The ARACNe network can be effectively interrogated (i.e., mined) using MARINa (Lefebvre et al., 2010) and VIPER (Alvarez et al., 2016), two algorithms that identify TFs/co-TFs as driver biomarkers associated with specific phenotypes (e.g., cancer initiation, cancer progression, metastasis, treatment response, etc.). Specifically, MARINa (Lim et al., 2009; Lefebvre et al., 2010) requires a differentially expressed signature, defined as a ranked list of genes between any two phenotypes of interest. Then, the activated and repressed targets for each TF/co-TF (as inferred by ARACNe) are assessed for their enrichment in the over- and under-expressed parts of this signature (Lefebvre et al., 2010; Figure 3). Such enrichment is referred to as TF/co-TF transcriptional activity, and if it is statistically significant, the TF/co-TF is referred to as a Master Regulator (MR). As a result of this analysis, a TF/co-TF is considered an “activated” MR if its activated targets are significantly enriched in the over-expressed part of the signature and/or its repressed targets are significantly enriched in the under-expressed part of the signature. Conversely, a “repressed” MR exhibits the opposite behavior. It is important to note that TF/co-TF transcriptional activity is not defined based on the differential expression of TFs/co-TFs themselves but instead on the differential expression of their transcriptional targets. This allows the identification of TFs/co-TFs that are not necessarily differentially expressed but are modified on the post-translational level and would otherwise be missed by traditional association methods.

Master Regulator Inference Algorithm has successfully identified MRs in various cancers, including prostate cancer (Aytes et al., 2014, 2018; Mitrofanova et al., 2015; Talos et al., 2017), breast cancer (Lim et al., 2009; Fletcher et al., 2013; Remo et al., 2015), pancreatic cancer (Sartor et al., 2014), ovarian cancer (Zhang et al., 2015), glioma (Carro et al., 2010; Sonabend et al., 2014), T cell acute lymphoblastic leukemia (Della Gatta et al., 2012), and diffuse large B cell lymphoma (Ying et al., 2013; Bisikirska et al., 2016). These biomarkers also serve as valuable therapeutic targets and their silencing could potentially have a significant effect on inhibition of malignant phenotype. To this extent, Mitrofanova et al. developed a computational algorithm to predict drug combinations that inhibit activity levels of FOXM1 and CENPF (MRs in malignant prostate cancer) and demonstrated that their therapeutic inhibition significantly improved cancer course (Mitrofanova et al., 2015). MARINa is freely available for download.^3^

At the same time, VIPER estimates TF/co-TF transcriptional activity on an individual sample-based level, as opposed to a two-phenotype signature-based level required by MARINa (Alvarez et al., 2016; Figure 3). In fact, while MARINa requires carefully selected multiple samples of the same phenotype to construct a differential expression signature, VIPER is able to utilize single-sample analysis by scaling the overall patient cohort (to its average expression for each gene). Furthermore, several advantages of VIPER include estimation of TF/co-TF activity through a so-called mode of regulation (taking into account whether targets are activated, repressed, or their direction cannot be determined), inference of regulator-target interaction confidence, and accounting for target overlap between different regulators (Alvarez et al., 2016). VIPER was shown to accurately infer aberrant oncoprotein activity induced by somatic mutations, across multiple cancer types (Alvarez et al., 2016). An R package is freely available.^4^

#### Multi-Omic Regulatory Network

Multi-omic data integration is another avenue to improve interpretability and discovery of functionally relevant biomarkers. Integration of different data modalities can increase the confidence of the overall findings since gene regulation is a complex process affected by multiple factors, such as gene mutations, structural variants, epigenomics, and more.

##### Network construction: RegNetDriver, step I

**FIGURE 4:**
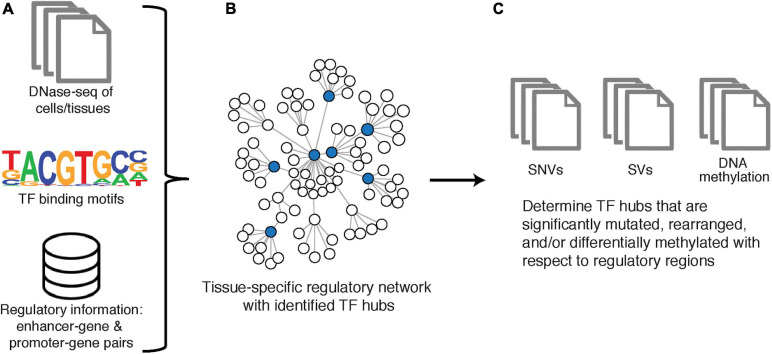
RegNetDriver. **(A)** DNase-seq of DNase I hypermutation sites from a specific tissue type, information to identify TFs from binding motifs, and information of known regulatory gene pairs as used as input to reconstruct **(B)** a tissue-specific regulatory network. TF hubs are determined from nodes with the top 25% out-degree centrality. **(C)** Significantly perturbed TF hubs are identified using SNV, SV, and DNA methylation data.

RegNetDriver is an algorithm for multi-omic tissue-specific regulatory network construction and analysis (Dhingra et al., 2017; Figure 4). The regulatory network reconstructed by RegNetDriver represents a two-layered relationship: (i) connecting TFs to promoter/enhancer regions; and (ii) further connecting promoter/enhancer regions to their corresponding target genes. To reconstruct relationships between TFs and promoters/enhancers of potential targets, Dhingra et al. utilize tissue-specific (i.e., prostate epithelium) DNase I hypersensitive sites to define accessible regulatory DNA regions and integrate this information with promoter/enhancer annotations from ENCODE (Encode Project Consortium, 2012) and GENCODE (Harrow et al., 2012). TFs are then connected to promoters/enhancers based on the enrichment of their binding motifs. Promoters/enhancers are further connected to their target genes through significant correlation of promoter/enhancer region activity signals (estimated using bisulfite sequencing and ChIP-seq data) with target gene expression profiles (estimated using RNA-seq data). Note that this is a directed two-layered network that estimates relationships between TFs and their transcriptional targets through their corresponding promoter/enhancer associations.

##### Network mining: RegNetDriver, step II

This network is then utilized to identify TF hubs with genomic and epigenomic alterations that can potentially cause large perturbations in this tissue-specific network. Specifically, TFs are first mined on degree centrality, such that the top 25% of TFs with the greatest number of outgoing edges are defined as hubs. Next, to identify TF hubs significantly affected on genomic and epigenomic levels in prostate cancer, they are evaluated for the presence of prostate-cancer specific genomic alterations (single nucleotide variants and structural variants) and DNA methylation changes in their coding and non-coding regulatory regions. In Dhingra et al., RegNetDriver nominated three TFs as regulatory drivers in prostate cancer, with functional validation conducted on *ERF* (Dhingra et al., 2017). RegNetDriver is freely available for download.^5^

### Protein–Protein Interaction Network-Based Analysis

**FIGURE 5:**
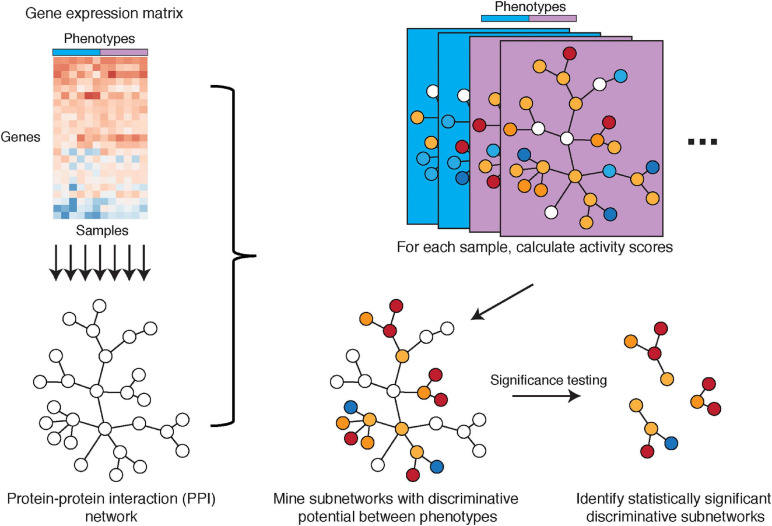
Illustration of the PPI network-based approach by Chuang et al. Gene expression microarray data with phenotype information is overlaid onto a PPI network that is constructed from existing knowledge. Subnetwork activities are calculated per sample based on z-transformed gene expression values, with subnetworks defined by the PPI network. Discriminative potential for each subnetwork is determined by mutual information (or alternatively, t-score or Wilcoxon score) that measures the association between sample activities and phenotypes. Subnetworks with discriminative potential between phenotypes are identified by a greedy search for locally maximal discriminative potential scores. Discriminative subnetworks are further assessed in significance testing to identify statistically significant discriminative subnetworks.

Another important avenue in mechanism-centric biomarker discovery is PPIs. Such interactions elucidate putative protein complexes, which are known to perform critical functions within the cell and include for example the pre-initiation complex for RNA transcription (Greber and Nogales, 2019), the spliceosome for pre-mRNA splicing (Chen et al., 2007), and the ribosome for translation of mRNA to protein (Wilson and Doudna Cate, 2012), among others. Cancer cells in particular have been shown to deregulate protein complexes for their sustained proliferation, survival, and metastasis (Robichaud et al., 2019). In recent years, numerous public databases have cataloged networks of known and predicted PPIs, such as STRING (Szklarczyk et al., 2019), IntAct (Orchard et al., 2014), CellCircuits (Mak et al., 2007), and PINA (Cowley et al., 2012) [more comprehensive lists are described by Huang et al. (2018) and Miryala et al. (2018)]. Here, we describe the method from Chuang et al. (2007), which effectively combines PPI networks with gene expression data and evaluates these hybrid subnetworks as mechanism-centric biomarkers of breast cancer metastasis (Figure 5).

#### Network Construction: Chuang et al., Step I

Chuang et al. introduce a hybrid approach to combine a PPI network with tissue-specific gene expression profiles across patient samples. The PPI network is comprised of nodes representing proteins and edges representing a characterized PPI, utilizing subnetworks from CellCircuits. Tissue-specific gene expression data are then overlaid onto all PPI subnetworks. For each subnetwork, its activity in each sample/patient is defined as a combination of z-scores for the subnetwork genes. This defines patient-specific vectors of subnetwork activities, which are then mined for phenotype associations.

#### Network Mining: Chuang et al., Step II

Activities of subnetworks are evaluated for their association with specific phenotypes (e.g., metastatic and non-metastatic), where associations can be calculated by mutual information, t-score, or Wilcoxon score and is referred to as the subnetwork discriminative potential/score. Next, the method selects subnetworks with a locally maximal discriminative score and performs significance testing to ensure subnetworks are non-random and robust. In classification performance on a test cohort, the authors found that the subnetwork markers identified using this PPI network-based approach showed higher AUC in classifying metastatic versus non-metastatic samples compared to single-gene markers, random subnetworks, and gene sets from other annotation databases such as GO and MSigDB. Importantly, the method by Chuang et al. showed better biomarker reproducibility (i.e., higher overlap between markers) between two different breast cancer studies, outperforming gene-centric methods (Chuang et al., 2007).

### Pathway-Based Analysis: pathCHEMO and pathER

Recently, pathway-based biomarker algorithms, such as pathCHEMO (Epsi et al., 2019) and pathER (Rahem et al., 2020), have demonstrated that discovery approaches that encompass information from biological pathways significantly outperform gene-centric methods which do not take into account pathway membership.

Pathways represent a group of biochemical entities (e.g., genes, proteins, etc.), connected by interactions, relations, and reactions (including physical interactions, complex formation, transcriptional regulation, etc.), that lead to a certain product or changes in a cell. Molecular pathways have long been known to play a crucial role in cancer initiation, progression, dissemination, and therapeutic response. Some notable examples are: the role of RAS and PI3K pathways in prostate and breast cancers and their therapeutic responses (Yue et al., 2002; Haagenson and Wu, 2010), the Wnt signaling pathway in colorectal and other cancers (Zhan et al., 2017), the Hippo pathway in melanoma (Zhang X. et al., 2020), and the MYC pathway in prostate cancer progression and treatment response (Arriaga et al., 2020).

**FIGURE 6:**
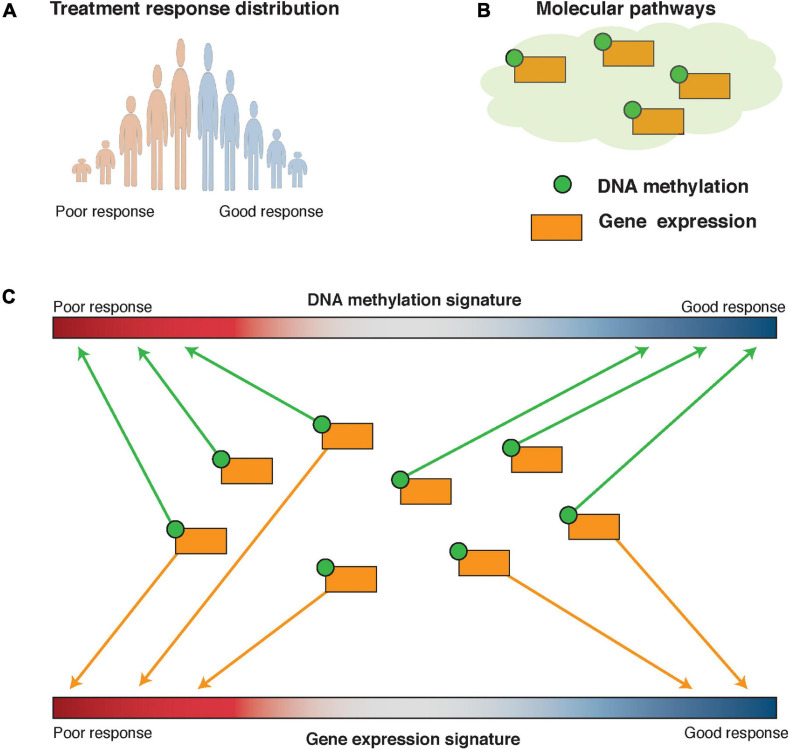
Pathway-based modeling: pathCHEMO and pathER. **(A)** Therapeutic response distribution is defined based on time to therapeutic failure. Tails of this distribution are utilized in pathCHEMO and a full spectrum of therapeutic responses is utilized in pathER. **(B)** Molecular pathways are utilized as a knowledge base in pathway-based modeling. Genes in such pathways can be affected on multiple levels, such as differential expression (i.e., orange square) and DNA methylation (i.e., green satellite). **(C)** Molecular pathways are assessed for their integrated enrichment and association with therapeutic response.

Both pathCHEMO and pathER assume that interrogation of molecular pathways, such as those present in Biocarta (Nishimura, 2001), KEGG (Kanehisa et al., 2021), and Reactome (Jassal et al., 2020), can reveal functional, biologically meaningful biomarkers that govern carcinogenesis and therapeutic response. pathCHEMO was specifically developed to compare poor versus good therapeutic response (as categorical outcomes) in cancer. In general, it evaluates differential behavior of biological pathways on both transcriptomic (RNA expression) and epigenomic (DNA methylation) levels between any two phenotypes of interest (Epsi et al., 2019). First, an RNA expression treatment response signature is defined as a list of genes ranked by their differential expression between poor and good treatment response. Then, genes in each pathway are evaluated for their enrichment in either over-expressed, under-expressed, or differentially expressed (which includes both over- and under-expressed) part of this signature. Enrichment in the over- and under-expressed parts separately allows identification of pathways where the majority of genes exhibit a similar behavior (i.e., are either over- or under-expressed), while enrichment in the differentially expressed part of the signature allows identification of pathways where some genes are over-expressed and some are under-expressed (which depicts a complex interplay of activation and repression relationships inside a molecular pathway). This enrichment is referred to as the RNA expression-based activity level of a molecular pathway. DNA methylation-based activity for each pathway is estimated in the same manner using a DNA methylation treatment response signature. Pathways that are enriched in the RNA expression treatment response signature and the DNA methylation treatment response signature are then integrated to select those that are significantly affected on both expression and methylation levels (Figure 6). Activity levels of the candidate pathways are further evaluated as biomarkers of therapeutic response in independent patient cohorts. Epsi et al. showed that pathCHEMO could successfully identify molecular pathways as biomarkers of response to commonly used chemotherapy in lung adenocarcinoma, lung squamous carcinoma, and colorectal adenocarcinoma (Epsi et al., 2019). Yet, a large number of genes that participate in these pathways could potentially preclude their adoption to clinic. To overcome this limitation, “read-out” genes for each pathway were identified for which expression levels (i) correlate with pathway activity and (ii) are associated with therapeutic response. Such read-out genes were shown to produce the same predictive accuracy as the pathways themselves and constitute feasible biomarkers for clinical use (Epsi et al., 2019). pathCHEMO is freely available at http://license.rutgers.edu/technologies/2019-121_pathchemo.

As opposed to pathCHEMO, pathER applies a pathway-based approach on a single-patient level, which allows the association of pathway activity across a patient cohort to a wide range of therapeutic responses (Rahem et al., 2020). Specifically, this approach utilizes a multivariable regression Cox proportional hazards model to associate pathway activity levels with time-to-therapeutic failure, thus capturing poor, good, and medium therapeutic responses. Rahem et al. successfully applied this approach to identify both pathways and their read-out genes for tamoxifen resistance in ER-positive breast cancer (Rahem et al., 2020). pathCHEMO and pathER were compared to other approaches, including black-box machine learning techniques (such as random forest and support vector machines) and differential gene expression alone, and were shown to outperform these approaches in identifying more accurate biomarkers of therapeutic response (Epsi et al., 2019; Rahem et al., 2020).

## Challenges and Limitations of Mechanism-Centric Approaches

Mechanism-centric approaches provide a powerful solution for informed biomarker discovery, yet common challenges that these methods need to account for include sufficient cohort sizes, data variability and scaling, comprehension of existing knowledge bases, and tissue-specificity (Table 1).

**TABLE 1:** Summary of mechanism-centric methods discussed in this review.

Method	Data modality	Utilize knowledge base?
**Gene co-expression network-based**	*Identify modules of highly correlated genes* +Increased interpretability at the mechanistic level +Associate genes with previously uncharacterized biological functions –Directionality of gene-gene interactions is unknown

Centered Concordance Index (CCI) (Han et al., 2016)	*Condition-specific module identification*
	Single-omic	No

Eigengenes (Alter et al., 2000; Zhang and Horvath, 2005)	*Identify modules associated with clinical features of interest*
	Single-omic	No

Hubs (Freeman, 1977; Horvath and Dong, 2008)	*Hub gene identification* +*Identify potential mechanism-centric target*
	Single-omic	No

**Regulatory network-based**	*Identify regulatory relationships between a TF/co-TF and its target genes* +Increased interpretability at the mechanistic level +Identify potential drivers of disease +Can identify non-linear relationships +Tissue specific network

MARINa (Lefebvre et al., 2010)	*Identify MRs from a set of samples containing two phenotypes* –Need phenotype signature
	Single-omic	No

VIPER (Alvarez et al., 2016)	*Single-sample MR identification from a cohort* –Dataset scaling
	Single-omic	No

RegNetDriver (Dhingra et al., 2017)	*Identify TF hubs that are significantly affected by single nucleotide variants, structural variants, or DNA methylation* +Increase interpretability of TF hub activity through multi-omic integration –Limited by information in knowledge base
	Multi-omic	Yes

**PPI network-based**	*Use PPI subnetworks as a functional unit* +Increased interpretability at the mechanistic level +Connect results to the protein complex level –Limited by information in knowledge base

Chuang et al., 2007	*Identify subnetworks with differential activity in metastatic breast cancer* +Tissue-specificity from overlaying gene expression data +Improved biomarker classification accuracy and reproducibility –Dataset scaling
	Multi-omic	Yes

**Pathway-based**	*Use molecular pathways as a functional unit* +Increased interpretability at the mechanistic level –Limited by information in knowledge base

pathCHEMO (Epsi et al., 2019)	*Identify significantly altered pathways (at transcript and DNA methylation levels) in response to chemotherapy in lung and colorectal cancer* +Improved biomarker classification accuracy and reproducibility –Need phenotype signature
	Multi-omic	Yes

pathER (Rahem et al., 2020)	*Identify pathways as markers of tamoxifen resistance in ER* + *breast cancer* +Improved biomarker classification accuracy and reproducibility –Dataset scaling
	Single-omic	Yes

*The objective of each method is detailed in italics, followed by their respective pros (+) and cons (–). Overall pros and cons for each method type are listed in a non-redundant manner. Information on data modality and if a method utilized a knowledge base is detailed as well.*

As many of these methods utilize association-based analyses (i.e., correlation, mutual information, regression, etc.), a sufficient cohort size is required to be able to accurately estimate relationships between variables. One of the direct solutions to this problem includes combining analyses in multiple datasets; however, batch effects among different acquisition methods, profiling platforms, and even institutions where datasets were collected might hamper such implementation.

In addition to a sufficient cohort size, substantial variability of expression profiles is also required to be able to accurately predict associations between variables. This task is feasible, yet it requires careful consideration, meticulous initial experimental design, and in-depth investigation of the amount of final variability necessary for successful analysis. Another challenge is the need for well-defined phenotypes, as they often require a substantially large number of samples inside each phenotype group while also demanding intra-sample homogeneity, as in the eigengene approach, MARINa, PPI network-based method by Chuang et al., pathCHEMO, etc.

At the same time, methods that rely on single-patient/sample mining (e.g., VIPER, the PPI network-based method by Chuang et al., and pathER) rely on dataset scaling to define its single-sample signatures (defined by comparing each gene to the average of its expression in the dataset of interest) making interpretation of any findings from such analyses dataset-specific.

Another known challenge is tissue-specificity, commonly faced in PPI network-based and pathway-based approaches, though some tissue- and cell-specific interaction databases are now available such as TissueNet (Basha et al., 2017), the Integrated Interactions Database (Kotlyar et al., 2019), and HumanBase (Greene et al., 2015). Tissue-specificity in these methods is usually achieved by overlaying gene expression data onto the PPI networks or molecular pathways, such as in Chuang et al., pathCHEMO, and pathER.

Furthermore, limitations of mechanism-centric approaches that utilize knowledge bases (e.g., RegNetDriver, PPI network-based approach, pathCHEMO, and pathER) lie in their reliance on known biological relationships among groups of genes/proteins/other functional units contained within a database. Various annotation, pathway, and PPI databases depend on existing information and do not include functional units that have not been previously studied, thus limiting *de novo* discoveries.

## Discussion

The wide availability of large-scale data produced by high-throughput technologies has created a wealth of information for biomarker discovery. A vast majority of these biomarkers have been identified using gene-centric methods, yet their interpretability and clinical utility have been limited as they do not account for the relationships among genes. Utilizing methods that consider biological underpinnings of the data (i.e., mechanism-centric methods) can vastly improve interpretable biomarker discovery, clinical applicability and targeting, and reproducibility of results.

In particular, advantages of mechanism-centric over gene-centric approaches can be illustrated through their ability to (i) identify a tightly connected, cooperative group of genes unified by the same function, as opposed to individual genes (which might not be related); (ii) provide a mechanism-level view, which enhances the understanding of the biological mechanisms implicated in a phenotype (e.g., therapeutic resistance, cancer metastasis); (iii) look at alterations in biological structures, which enhances the likelihood of identifying functionally relevant targets; (iv) identify driver as opposed to passenger markers, which allows for their effective therapeutic targeting; (v) focus on molecular structures, rather than individual genes, which decreases the chance of detecting results due to experimental noise present in biological experiments (i.e., robustness of results); and finally (vi) identify biomarkers that are more accurate and more reproducible between different cohorts.

From a computational point of view, mechanism-centric approaches can be used for interpretable feature engineering and selection (i.e., reduction), subsequently reducing the number of hypotheses to be tested. This is clearly demonstrated by gene co-expression networks, regulatory networks, PPI networks, and pathway-based methods, where cooperative groups of genes, instead of a long list of singular genes, are assessed for their association with clinical outcomes.

Mechanism-centric methods can both (i) provide interpretable inputs to white- or black-box approaches or (ii) contribute to inner model interpretability (i.e., such as in visible machine learning). First, results from mechanism-centric methods can be utilized as inputs into learning models to significantly improve predictive performance (over gene-centric inputs). One such example was demonstrated in Rahem et al., where pathway-based markers were utilized as inputs into Cox proportional hazards regression modeling and outperformed gene-centric markers for tamoxifen resistance in ER-positive breast cancer (Rahem et al., 2020). Similarly, Chuang et al. showed that markers identified by their PPI network-based method could be effectively used as inputs into a regression model and outperformed gene-centric markers in classification of metastatic breast cancer (Chuang et al., 2007). Though not in cancer, several methods have also suggested utilizing hierarchical structures (such as those inherent in Gene Ontology) as inputs for predictive models (Carvunis and Ideker, 2014; Yu et al., 2016). Second, mechanism-centric methods can potentially be incorporated into model building, such as in “visible learning,” where the relationships between inputs and outputs can be interpreted (Yu et al., 2018). One such (outside of cancer) neural network method, DCell, was proposed by Ma et al., where the hierarchy of molecular relationships determined from prior knowledge (Gene Ontology and CliXO) was built into the model itself (i.e., hierarchies were utilized by nodes of the neural network) (Ma et al., 2018). Recently, Kuenzi et al. developed an extension of DCell, called DrugCell, which utilized chemical drug structures as a part of the neural network learning model to predict drug response in cancer cells (Kuenzi et al., 2020). This interpretable deep learning model was shown to be able to predict cell sensitivity/resistance to specific drugs, synergistic drug mechanisms, and effective drug combinations for treatment.

Further improvements in the interpretability of biological processes that inform discovery of mechanism-centric biomarkers can be made through multi-level data and method integration. For example, several groups have combined co-expression WGCNA modules with PPI networks to uncover hubs with functional connections as biomarkers in endometrial cancer (Liu et al., 2019) and bladder cancer (Wang Y. et al., 2019). Wang et al. constructed an Active Protein-Gene network model using transcriptional regulatory and PPI networks to quantify TF activity and elucidate both upstream and downstream regulations (Wang et al., 2013). Even though this study was done in diabetes, it could be applicable to mechanism-centric biomarker discovery in cancer. Ahsen et al. embedded VIPER within a new framework (NeTFactor) to identify TFs that most likely regulate a gene-centric biomarker signature (Ahsen et al., 2019). While this method was applied to asthma and peanut allergy, it could easily be extended to cancer studies. At the same time, multi-omic integration in RegNetDriver improved the interpretability of the proposed model to explain the impact of mutations, structural variants, and DNA methylation on TF activity in prostate cancer (Dhingra et al., 2017). A recent study by Broyde et al. constructed a multi-omic lung adenocarcinoma tissue-specific oncoprotein interaction network using information obtained from ARACNe, CINDy (an algorithm identifying post-translational modulators), VIPER, and PPI predictions (Broyde et al., 2021), which depicted a complex network of interactions for KRAS and could potentially be utilized for mechanism-centric biomarker discovery. Such multi-level approaches in conjunction with mechanism-centric methods promise to uncover a deeper understanding of mechanisms involved in gene regulation and post-translational modifications in biomarker discovery.

Finally, recent technological advances, such as those seen in single-cell studies, promise to improve our understanding of intra-tumor heterogeneity, clonal evolution, and the role of microenvironment in cancer progression and therapeutic response. Single-cell gene expression offers a granular view of active pathways in a cell type-specific manner and potentially allows for the construction of cell type-specific networks. In fact, the rapid advances of single-cell sequencing technology have already allowed network analysis methods to be applied directly to data from single-cell RNA-sequencing (scRNA-seq) (Crow et al., 2016; Aibar et al., 2017; Chan et al., 2017; Fiers et al., 2018; Papili Gao et al., 2018; van Dijk et al., 2018; Lamere and Li, 2019; Jackson et al., 2020; Sekula et al., 2020; Ye et al., 2020) with integration of other data modalities for improved network inference (Aibar et al., 2017; Chan et al., 2017; Papili Gao et al., 2018; van Dijk et al., 2018; Jackson et al., 2020; Pratapa et al., 2020). Furthermore, matching single-cell and bulk patient samples could provide an invaluable resource for single-cell driven network investigations that can be compared to and related back to bulk tissues. As more single-cell data become available (e.g., RNA sequencing, targeted DNA sequencing, ATAC-seq, etc.), we foresee advances in single-cell technologies and data analysis to be central to understanding precise, clone-specific biomarkers, unveiling trajectories of tumor evolution and providing accurate ground for informed time-cautious precision therapeutics.

In summary, mechanism-centric approaches (based on gene co-expression networks, regulatory networks, PPI networks, and molecular pathways) identify biomarkers that are biologically meaningful, interpretable, reproducible, have higher translational potential, and provide greater predictive power over biomarkers identified by gene-centric methods. Thus, mechanism-centric approaches are the future of clinically relevant rational biomarker discovery, personalized treatment planning, and precision therapeutics in cancer.

## Author Contributions

CY and AM conceived and wrote the manuscript. Both the authors contributed to the article and approved the submitted version.

## Conflict of Interest

The authors declare that the research was conducted in the absence of any commercial or financial relationships that could be construed as a potential conflict of interest.

## Publisher’s Note

All claims expressed in this article are solely those of the authors and do not necessarily represent those of their affiliated organizations, or those of the publisher, the editors and the reviewers. Any product that may be evaluated in this article, or claim that may be made by its manufacturer, is not guaranteed or endorsed by the publisher.

## Footnotes

1
https://cran.r-project.org/package=lmQCM

2
http://califano.c2b2.columbia.edu/aracne

3
http://califano.c2b2.columbia.edu/marina

4
http://doi.org/10.18129/B9.bioc.viper

5
https://khuranalab.med.cornell.edu/RegNetDriver.html
